# Extracellular vesicles: new targets for vaccines against helminth parasites

**DOI:** 10.1016/j.ijpara.2020.04.011

**Published:** 2020-08

**Authors:** Claire Drurey, Gillian Coakley, Rick M. Maizels

**Affiliations:** a Wellcome Centre for Integrative Parasitology, Institute of Infection, Immunity and Inflammation, University of Glasgow, 120 University Place, Glasgow G12 8TA, UK; b Department of Immunology and Pathology, Central Clinical School, Monash University, 89 Commercial Road, Melbourne, Victoria 3004, Australia

**Keywords:** Helminth, Extracellular vesicle, Vaccine, Exosomes, Parasite antigen

## Abstract

The hunt for effective vaccines against the major helminth diseases of humans has yet to bear fruit despite much effort over several decades. No individual parasite antigen has proved to elicit full protective immunity, suggesting that combinatorial strategies may be required. Recently it has been discovered that extracellular vesicles released by parasitic helminths contain multiple potential immune modulators, which could together be targeted by a future vaccine. Increasing knowledge of helminth extracellular vesicle components, both enclosed by and exposed on the membrane, will open up a new field of targets for an effective vaccine. This review discusses the interactions between helminth extracellular vesicles and the immune system discovered thus far, and the advantages of targeting these lipid-bound packages with a vaccine. In addition, we also comment upon specific antigens that may be the best targets for an anti-helminth vaccine. In the future, extensive knowledge of the parasites' full arsenal in controlling their host may finally provide us with the ideal target for a fully effective vaccine.

## Highlights

• Current vaccine candidates against helminth infection have shown limited success.
• Helminths release extracellular vesicles (EVs) which act on host cells and are a rich source of antigens for new vaccines.
• The biogenesis, release and immunomodulatory functions of helminth EVs are reviewed.
• Utilisation of EVs in vaccine generation are discussed, including potential antigens and routes of delivery.

## Graphical abstract

**Figure:**
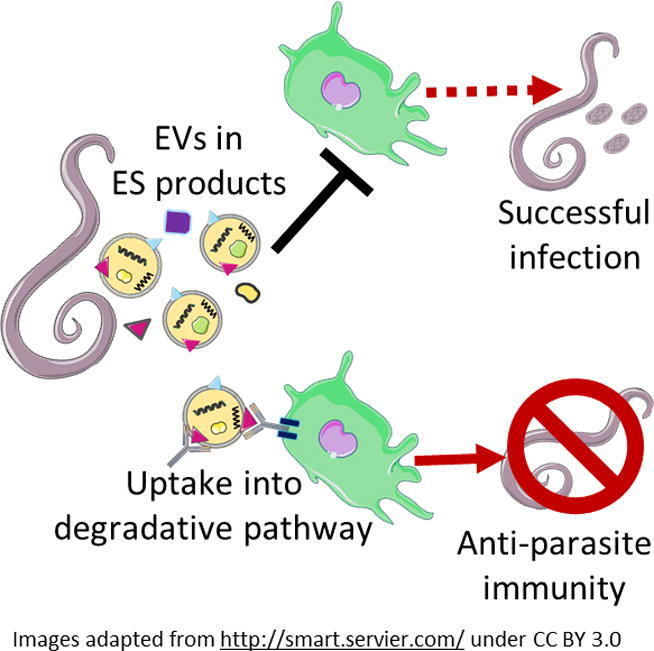

## 1 Introduction

### 1.1 Helminth infection and why we need a vaccine

Helminth infections are widely spread around the globe and cause a spectrum of neglected tropical diseases. More than 1.5 billion people, or 24% of the world's population, are infected with soil-transmitted helminths alone (Pullan et al., 2014, Jourdan et al., 2018). In addition, with 142 million people affected by schistosomiasis (James et al., 2018) and 87 million by the vector-borne helminthiases of lymphatic filariasis and onchocerciasis (James et al., 2018) it is undeniable that helminth parasites are a huge global disease burden. Although often overlooked due to a lack of direct mortalities, it is increasingly recognised that helminth infection can have many wide-ranging morbidities including anaemia and growth stunting, cognitive defects, elephantiasis, blindness and nodding syndrome/epilepsy, and in some cases cancer (Mostafa et al., 1999, Bethony et al., 2006, Sripa et al., 2007, Hotez et al., 2008, Shang et al., 2010, Pullan et al., 2014, Johnson et al., 2017, Pabalan et al., 2018). Infection with helminths affects the poorest and most deprived communities and is a health challenge we have yet to solve due to the lack of a vaccine (Hewitson and Maizels, 2014, Diemert et al., 2018), and the rapid rate of reinfection after treatment (Jia et al., 2012).

In addition to human infection, parasitic helminth infection of farmed animals also causes significant economic losses across the world and provides the potential for transmission of zoonotic diseases (Robinson and Dalton, 2009, Charlier et al., 2014). Widespread use of anthelmintics in farming has led to the rapid evolution of drug resistance in some livestock-prevalent species (Woodgate et al., 2017), suggesting that resistance might also become a problem for human disease in the not too distant future (Vercruysse et al., 2011, Sangster et al., 2018). Development of a vaccine would be the ideal solution as repeated treatments would not be needed, and if composed of a combination of antigens, the parasite could not evolve resistance in the same way as to a drug. Furthermore, vaccines against helminths of livestock with irradiated larvae have proven successful (reviewed by Hewitson and Maizels (2014)), and the barriers to testing animal vaccines are less formidable than with humans; hence control of parasites in animals remains a high priority.

Overall, the pathway to a successful helminth vaccine has proven difficult to negotiate; many antigens have been identified in animal models, although typically able to reduce worm burdens by 25–50% rather than achieving sterilising immunity. Few of these have progressed to evaluation in human trials, which first require a raft of refinement and regulatory steps to be fulfilled (Diemert et al., 2018). Even vaccines advancing to the trial stage may encounter obstacles. The *Necator americanus* vaccine candidate, *Ancylostoma* Secreted Protein-2 (*Na-*ASP-2), initially seemed promising but led to generalised urticarial reactions in some participants of a phase 1b trial in a *Necator*-endemic area, believed to be due to immunoglobulin E (IgE) against Na-ASP-2 from previous infections (Diemert et al., 2012). In its place, two further hookworm vaccine candidates are being evaluated, glutathione S-transferase 1 (*Na-*GST-1), an enzyme found in the blood-feeding parasite gut involved in detoxification of free heme (Zhan et al., 2010), and another digestive enzyme, aspartic protease-1 (*Na*-APR-1) (Seid et al., 2015). Phase 1 trials have been less problematic and might lead the way for a combined vaccine formulation (Diemert et al., 2017). Several candidates for a schistosome vaccine are also in human trials (Molehin et al., 2016), including the membrane surface proteins tetraspanin (Sm-TSP-2) and calpain (Sm-p80) (Siddiqui and Siddiqui, 2017), in studies that have recently been well reviewed (Stutzer et al., 2018).

Parasitic helminths of medical and veterinary concern comprise two distant phyla; the nematoda (roundworms) and the platyhelminths (flatworms). The flatworms can further be divided into cestodes (tapeworms) and trematodes (flukes). The association between host and parasite is an intimate one, and in many cases can last for a long time. Hookworms live in the human intestine between 1 and 3 years for *Ancylostoma duodenale* and 3–10 years for *Necator americanus* (Hoagland and Schad, 1978, Brooker et al., 2004), although a maximum lifespan of 18 years has been demonstrated (Beaver, 1988). Some tapeworms can even survive for 20–30 years (Sandground, 1936). Although nematodes and platyhelminths are evolutionarily distant, the parasites occupy similar niches within their hosts and have developed similar methods of host immune manipulation to ensure long-term survival and to reproduce abundantly (Maizels and McSorley, 2016).

Host manipulation by helminths is primarily due to the release of molecules into their environment, broadly referred to as excretory/secretory (ES) products (Lightowlers and Rickard, 1988, Maizels et al., 2018). These components from parasites are known to have immunomodulatory properties, for instance ES product from the model mouse nematode *Heligmosomoides polygyrus* (HES) is able to suppress allergic responses (McSorley et al., 2015), modulate dendritic cells (Segura et al., 2007), and induce regulatory T cells (Grainger et al., 2010). Similar to many helminth products, ES products from the trematode *Fasciola hepatica* skews the immune response from Th1 to Th2 (Robinson et al., 2013), as does ES product from the nematode *Nippostrongylus brasiliensis* (Balic et al., 2004), and proteins released from schistosome eggs (Everts et al., 2009), in both cases acting through dendritic cells. These and many similar studies (reviewed in Harnett, 2014, Maizels et al., 2018) have been accompanied by detailed proteomic (Ditgen et al., 2014) and glycomic (Hokke and van Diepen, 2017) analyses of the composition of ES materials, extending also to small RNAs (Britton et al., 2014), and perhaps most unexpectedly, extracellular vesicles (EVs).

The finding that parasites produce and release EVs was first demonstrated in the trematode parasites *Echinostoma caproni* and *F. hepatica* (Marcilla et al., 2012). EVs were seen by transmission electron microscopy (TEM) after ultracentrifugation of ES materials from both trematodes, and subsequent analysis by mass spectrometry identified 45 and 79 different proteins in these EVs respectively (Marcilla et al., 2012). Nematode and cestode parasite ES products have also been found to contain EVs (Buck et al., 2014, Hansen et al., 2015, Zamanian et al., 2015, Tzelos et al., 2016, Ancarola et al., 2017). Investigation has revealed that EVs are a common feature of parasite secretions across a wide range of species (summarised by Coakley et al., 2015, Eichenberger et al., 2018b, Tritten and Geary, 2018).

### 1.2 What is under the EV umbrella?

**Fig. 1:**
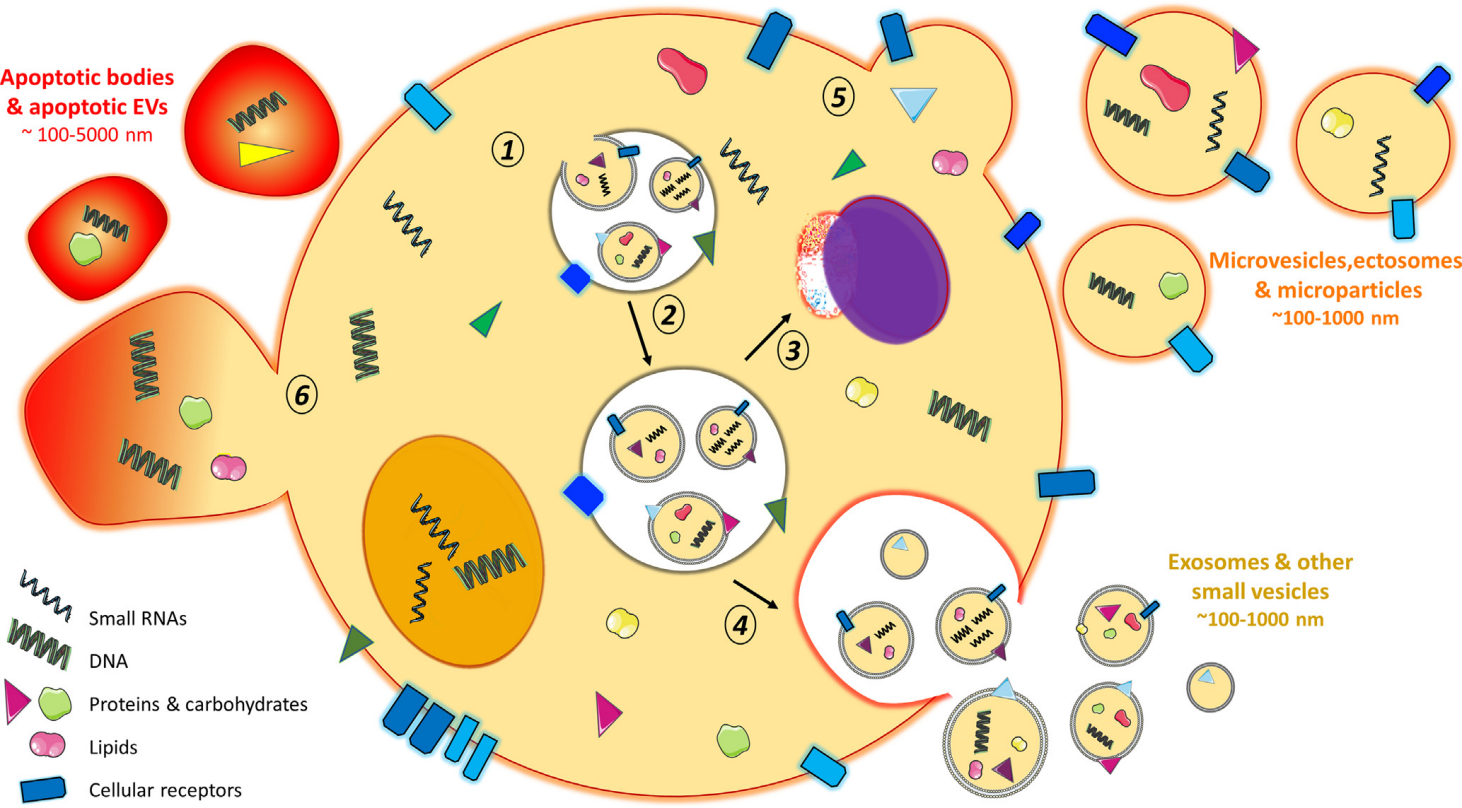
Overview of specific vesicle biogenesis and secretion pathways. Intra-luminal vesicles (ILVs) are formed within early endosomes via inward budding, retaining membrane proteins, lipids and other cytosolic contents of the parent cell (1). Endosomes mature to become late endosomes/multivesicular bodies (MVBs) (2) and degrade their contents via fusion with the lysosome (3) or release their ILVs into the extracellular environment (where they are now classed as “exosomes”) following fusion with the plasma membrane (4). Other methods of secretion include ‘budding’ of larger vesicles, such as microparticles, microvesicles and ectosomes, directly from the plasma membrane (5) or following programmed cellular death, whereby vesicles known as apoptotic bodies “bleb” from the cellular surface (6). Images are adapted from Servier Medical Art by Servier (http://smart.servier.com/) and modified by the authors under the following terms: Creative Commons Attribution 3.0 Unported (CC BY 3.0).

EVs are particles released from a cell that are delimited by a lipid bilayer, which shields the contents from enzymatic degradation that would occur in the extracellular environment. Originally, EVs were identified as large vesicles up to 5000 nm released during apoptosis, termed apoptotic bodies (Hristov et al., 2004), but it was subsequently found that healthy cells also release vesicles into their extracellular environment (Raposo and Stoorvogel, 2013). Broadly speaking, EVs can be classified into exosomes, ectosomes (microvesicles/microparticles) and apoptotic bodies according to their physical appearance, cellular and subcellular origins, and biochemical composition (Fig. 1). Exosomes are 50–100 nm diameter vesicles that are released by multivesicular late endosome fusion with the plasma membrane, whereas secreted ectosomes and microvesicles are 100–1000 nm vesicles that result from direct outward budding from the plasma membrane (Akers et al., 2013, Mathieu et al., 2019). Within parasite secretions, EVs the size of both exosomes and ectosomes have been identified (Cwiklinski et al., 2015). As it can sometimes be difficult to differentiate the two from each other, for instance in determining their subcellular origin specific to each parasite, both have been studied in the context of host-parasite interactions under the umbrella term “extracellular vesicle”, or EV (Marcilla et al., 2014, Coakley et al., 2015, Evans-Osses et al., 2015). EV compositional analysis can shed some light on their biogenesis. As exosomes go through the endosomal pathway, they are often associated with proteins of the Endosomal Sorting Complexes Required for Transport (ESCRT) pathway; in contrast, microvesicles which have directly budded from the plasma membrane contain many of the same markers as their parental external membrane.

### 1.3 Helminth EVs – whence?

Whether helminth EVs fall into this neat dichotomy, or are produced by other routes, remains to be determined. Tracking the exact site of secretion can be a challenging task, involving TEM localisation of vesicles and mass spectrometry analysis of their contents, matching this where possible to the protein profile of a potential secretion site. In particular, it is important to note a fundamental difference between the platyhelminths, with a brush border-like external lipid-rich membranous tegument, and the nematodes with an acellular cuticle formed of rigidly cross-linked collagens and other matrix proteins.

In the case of flatworms such as *F. hepatica*, the tegument is a major source of EVs, with a smaller exosome-like population shed from the tegument of the adult fluke (Cwiklinski et al., 2015, de la Torre-Escudero et al., 2016), some of which are seen in multivesicular bodies, suggesting a classification as exosomes. EVs are also released by *F. hepatica* from specialised cells lining the parasite gastrodermus (Cwiklinski et al., 2015, de la Torre-Escudero et al., 2019). Similarly, TEM has shown EVs at the tegument surface of the trematodes *E. caproni*, *Dicrocoelium dendriticum* and of *Schistosoma mansoni* cercariae (Samuelson and Caulfield, 1985, Marcilla et al., 2012, Bernal et al., 2014, Coakley et al., 2015).

Among the nematodes, the pattern appears to follow secretion from an internal site before release with fluids through a body opening. TEM of *H. polygyrus* adult worm intestine identified vesicle budding of the same size as exosomes, while proteomic analysis identified a suite of EV components corresponding to worm intestinal proteins on the apical surface of the epithelium. In addition, Scanning Electron Microscopy (SEM) of the anterior opening of *H. polygyrus* visualised structures similar in size to exosomes (Buck et al., 2014, Coakley et al., 2015). High levels of plasmalogen lipid components were found in *H. polygyrus* EVs compared with EVs from its murine host. While these correlate with an increased rigidity that may enable longevity in the intestine, they also indicated a specialised source, yet to be localised, at which EVs are selectively generated (Simbari et al., 2016).

**Fig. 2:**
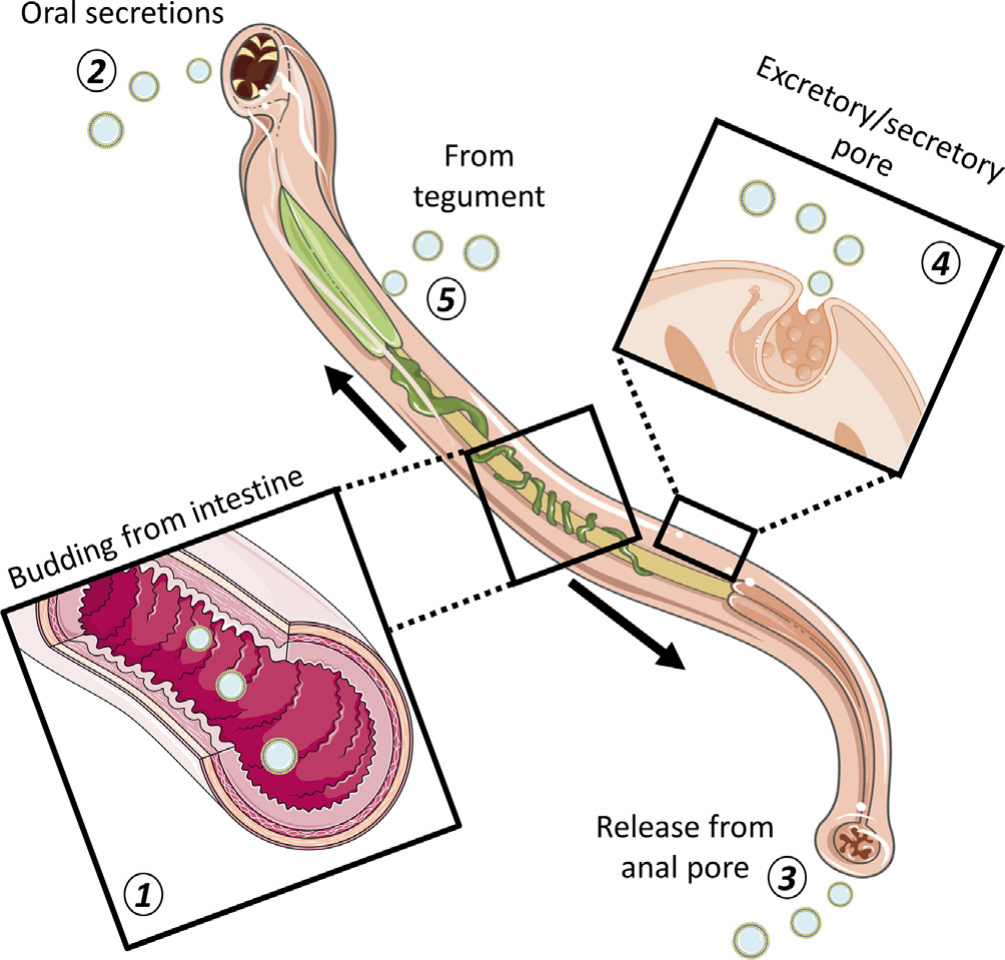
Vesicle release from helminth parasites. Both platyhelminths and nematodes have been found to release extracellular vesicles (EVs) from the gastrodermis (1). In nematodes, EVs released in the intestines may be released into the host via the anterior (2) or posterior (3) openings. EVs of *Brugia malayi* have also been found to be secreted from the excretory/secretory pore (4). In the case of platyhelminths, EVs can be shed directly from the tegument itself into the surrounding environment (5). Images are adapted from Servier Medical Art by Servier (http://smart.servier.com/) and modified by the authors under the following terms: Creative Commons Attribution 3.0 Unported (CC BY 3.0).

In other studies of nematodes, the ES pore of *B. malayi* microfilariae was found to be a candidate site for EV release; fluorescent labelling of a homolog to the ESCRT-associated protein Alix (ALG-2-interacting protein X), which is used as an exosome marker, showed staining focused around the pore and its associated duct (Harischandra et al., 2018). Investigations in *Ascaris suum* could not distinguish between EV secretion from the intestinal lining followed by exit via the anal pore, or EVs originating from the body fluid and exiting through the secretory pore, although similarity of microRNA (miRNA) composition suggests that both sites contribute (Hansen et al., 2019). Indeed, EVs may be released from different sites of a parasite throughout its lifecycle, depending on what needs to be secreted and the site of interaction with its host (Fig. 2).

### 1.4 Helminth EVs – function?

The characterised function of EVs is in intercellular communication, with regulation of multiple cellular processes including cell proliferation, survival and transformation (Maas et al., 2017, van Niel et al., 2018). Vesicles from infectious agents have also been identified as a means of intra-species communication, with functions being found in protozoan parasites, including species of *Plasmodium* and *Trypanosoma* (Mantel et al., 2013, Wu et al., 2018). The identification of EVs in helminth parasite secretions therefore suggests a role in manipulation of the host. There is growing evidence across multiple systems for parasite EV uptake by host cells. In the case of parasites dwelling in the intestinal tract, EVs from the trematode *E. caproni* and the nematode *H. polygyrus* are internalised by intestinal epithelial cells (Buck et al., 2014, Marcilla et al., 2014). Moreover, the intestinal nematode parasites *Trichuris muris* and *N. brasiliensis* both produce EVs which are actively taken up by mouse colonic and small intestinal organoids, respectively (Eichenberger et al., 2018a, Eichenberger et al., 2018c). Human cholangiocytes, the cells of the bile duct epithelium, have been shown to take up EVs secreted by the liver fluke *Opisthorchis viverrini* (Chaiyadet et al., 2019). These cell types are those that would be in direct contact with the parasitic helminth, so would be close to the site of EV release in vivo and an ideal target for regulating their surrounding environment.

Other parasite species, and invasive larvae in the life cycles of some intestinal parasites, may also target cells of the immune system to render an environment favourable to survival. In fact, *H. polygyrus, B. malayi* and *F. hepatica* EVs are all taken up by murine macrophages (Zamanian et al., 2015, Coakley et al., 2017, Harischandra et al., 2018, de la Torre-Escudero et al., 2019). EVs from the tissue-dwelling metacestode stage of *Echinococcus granulosus* are internalised by murine dendritic cells (Nicolao et al., 2019). This suggests that not only are helminths capable of shaping their immediate environment via the release of EVs, but the broader immunological status of their host too.

Across the whole field of EV research, the mechanisms of EV uptake and cargo delivery into the cytosol of target cells remains incompletely characterized (Mathieu et al., 2019). The first step involves targeting the acceptor cell, although whether this requires a specific combination of proteins or lipids on either the EV surface or acceptor cell, is unresolved (Mathieu et al., 2019). The internalisation of parasite EVs by cell types that engage in active endocytosis for their function, such as macrophages and dendritic cells, suggest that uptake might be stochastic (Zamanian et al., 2015, Coakley et al., 2017, Harischandra et al., 2018, Nicolao et al., 2019). The lack of preference in uptake of EVs from different life stages of *B. malayi* by murine macrophages despite differences in cargo supports this (Harischandra et al., 2018). However, there is still the possibility that molecules found on the exterior of EVs might facilitate interaction with specific cell types. Investigation of surface-exposed lectins on *F. hepatica* EVs found that they were distinct from those on the parasites surface and treatment of EVs with a glycosidase blocked uptake by macrophages (de la Torre-Escudero et al., 2019).

**Fig. 3:**
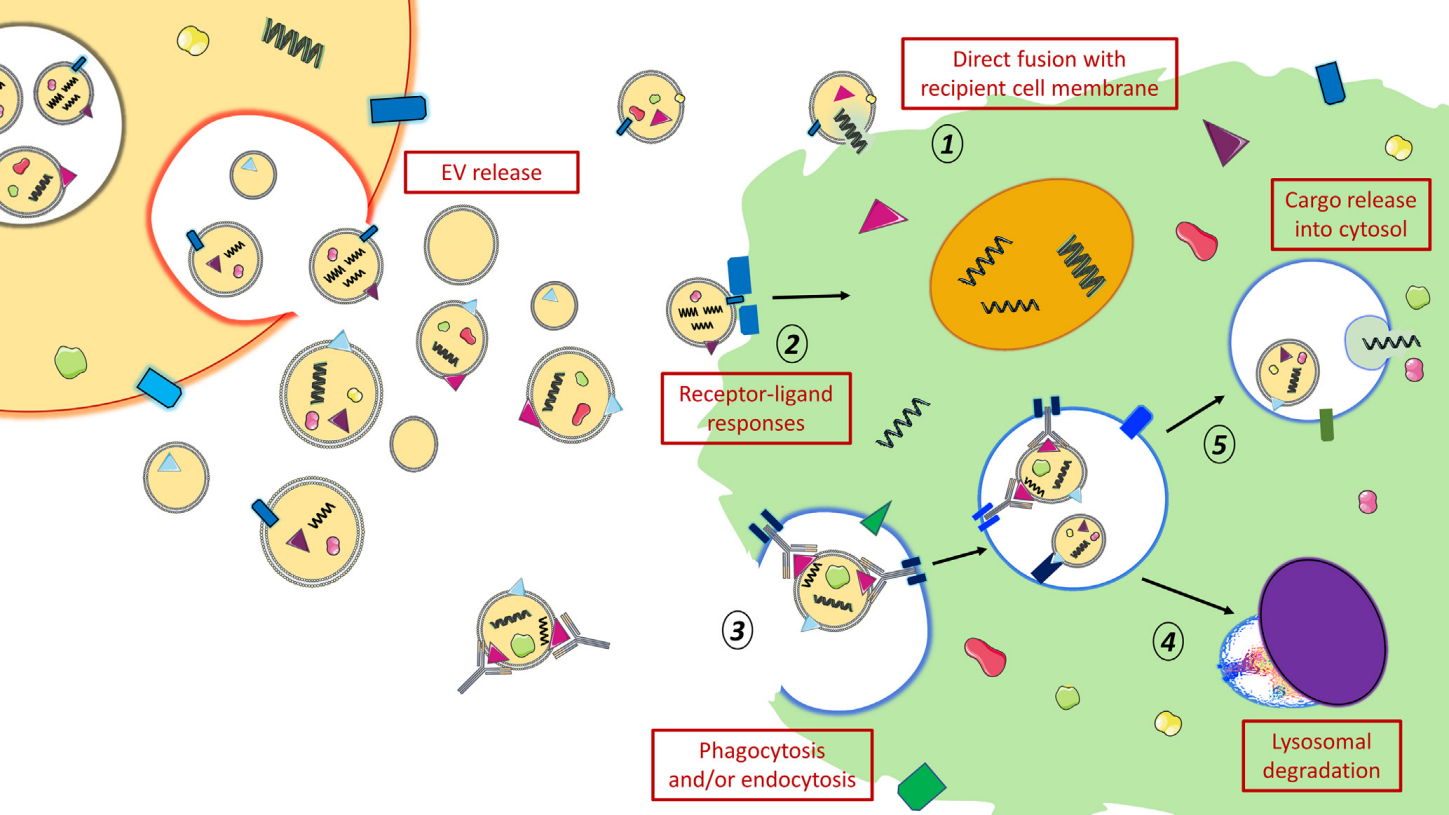
Proposed methods of exosome uptake. Exosomes can generate numerous responses in recipient cells, and are suggested to do so through at least three different mechanisms. Exosomes and other vesicles might directly fuse to the plasma membrane of the recipient cell, although the biological pathways involved in this are still poorly understood (1). Exosomes might also directly target receptors on the exterior surface of the recipient cell, driving host responses e.g. by co-stimulation through receptor-ligand interactions (2). Exosomes are also known to be taken up by recipient cells by phagocytosis, macro/micropinocytosis or endocytosis (caveolin/clathrin-dependent, receptor or antibody-mediated) (3). From our studies, we showed that antibodies enhance uptake of extracellular vesicles (EVs) into recipient cells, which are subsequently targeted for lysosomal degradation (4). Alternatively, internalised exosomes and other vesicles might utilise endosomal escape to release their contents directly or indirectly into the recipient cell cytosol (5). Images are adapted from Servier Medical Art by Servier (http://smart.servier.com/) and modified by the authors under the following terms: Creative Commons Attribution 3.0 Unported (CC BY 3.0).

After contact with target cells, uptake of EVs is believed to occur through endocytosis, with both clathrin-dependent and independent pathways having been reported (Mulcahy et al., 2014, Zakeri et al., 2018, Mathieu et al., 2019), as shown in Fig. 3. Uptake of helminth parasite EVs appears to be an active process, as uptake of *T. muris* EVs in colonic organoids kept at 4 °C did not show internalization (Eichenberger et al., 2018c). Uptake of EVs from *O. viverrini* by cholangiocytes could be blocked using antiserum to membrane tetraspanin proteins (Chaiyadet et al., 2019). *Brugia malayi* EVs have been found to be taken up by murine macrophages via phagocytosis (Harischandra et al., 2018). Cytochalasin D treatment, which inhibits endocytosis and phagocytosis by interfering with actin polymerisation, prevented the uptake of *H. polygyrus* EVs in bone marrow-derived macrophages (Coakley et al., 2017). Therefore, some form of receptor signalling can be presumed to be required.

It is speculated that once taken up, parasite EVs can either be internalized and targeted to the lysosome for degradation or recycled for release back to the extracellular space (Luga et al., 2012, Mathieu et al., 2019). However, it should not be discounted that parasite EVs could also act at the cell surface without delivery of their content; during immune responses EVs from dendritic cells containing Major Histocompatibility Complex (MHC)-peptide complexes can activate T-cells (Tkach et al., 2017). Many proteins identified within EVs contain transmembrane domains, so might have a function on the exterior of the EV in this way. EVs could also be a means of communication between different individuals of the same species within a host, or between different parasites in the same host.

### 1.5 Helminth EVs – the cargo

To understand the function of parasite EVs, it is vital to identify their contents, which may include proteins, RNA and lipids. Most data currently available is at the proteomic level, summarised in a recent review (Mekonnen et al., 2018). In addition, detailed analyses of small RNA profiles in EVs have been carried out for some species (Buck et al., 2014, Zamanian et al., 2015, Zhu et al., 2016). A specific nematode argonaute protein has been found that is delivered with small interfering RNAs (siRNAs), suggested to provide selectivity in small RNA packaging into EVs, although it might also play a role in facilitating endogenous gene regulation in the host after uptake (Chow et al., 2019).

Not only can proteins and RNAs be packaged in exosomes in order to have an effect on the host, EVs are also a means of lipid transport between cells, and they are known to transport free fatty acids and prostaglandins, as well as enzymes involved in their synthesis (Subra et al., 2010). EVs carrying bioactive lipids are important in immune system function (Sagini et al., 2018). *Schistosoma mansoni* lipids have been identified that activate eosinophils (Magalhaes et al., 2018) and it has been argued that an effective way for this lipid delivery to occur could be via EVs (Coakley et al., 2019).

EV contents may change throughout a parasite's lifecycle. Analysis of mRNA found in EVs from different life stages of *Haemonchus contortus* suggest that the gut is the likely source of vesicle-associated miRNAs in the L4 stage but not in the adult worm (Gu et al., 2017). EVs from *B. malayi* are produced across all stages of the lifecycle, right through from microfilariae to adult male and female worms (Harischandra et al., 2018), although EVs are most abundantly released from L3s, with a lower concentration in adult secretions (Zamanian et al., 2015). *Ascaria suum* contains more unique miRNAs in L3 and L4 EVs compared with adult-produced EVs (Hansen et al., 2019). These differences may be due to L3s needing to manipulate the host much more during migration through different tissues, compared with adults that have fixed residence in the lymphatics and small intestine respectively.

Harischandra et al. (2018) found that the EV proteome of *B. malayi* is both stage- and sex-specific, with 74 proteins identified in female EVs compared with only 20 for male worms (Harischandra et al., 2018). This is similar to the ES products of *B. malayi* that are already known, where female parasites secrete a greater number of proteins, and in greater amounts, than male worms (Moreno and Geary, 2008, Bennuru et al., 2009). Proteins with putative immunomodulatory functions, such as BmMIF-1, were enriched in the female worm EV profile, suggesting a more immunomodulatory role (Harischandra et al., 2018). Perhaps the close association of males and females in the lymphatic duct renders the production of these proteins by the males obsolete.

## 2 Immunosuppression by helminth EVs

The contents of parasite EVs, such as small RNAs matching host targets, and uptake by immune cells, suggest some role in immunomodulation of their respective hosts. Indeed, there is growing evidence that uptake of EVs from helminth parasites leads to downregulation of immune responses. Examples of this are presented in Table 1. The majority of uptake and effects reported so far are for innate immune cells; mainly macrophages, which tend to uptake extracellular contents (Wang et al., 2015, Zamanian et al., 2015, Coakley et al., 2017). It is interesting to see EV effects on cells that are directly in contact with the parasite itself. Investigation of the effect of *O. viverrini* EVs on human cholangiocytes found that they drove cell proliferation and induced changes in proteins associated with cancer pathways, suggesting a contribution to the development of cholangiocarcinoma in liver fluke-infected patients (Chaiyadet et al., 2015). Uptake by epithelial cells has been proven (Buck et al., 2014, Eichenberger et al., 2018a, Eichenberger et al., 2018c), and Buck et al. (2014) found downregulation of genes by EVs in a mouse epithelial cell line, including a regulator of MAPK signalling and a subunit of the IL-33 receptor. Investigation of a wider range of cell types may help to explain more of the pathological sequelae of helminth parasite infection.

**Table 1:** Immunological effects of helminth extracellular vesicles (EVs), in vitro and in vivo.

Parasite	Model used	Cell type	Action	Protein/RNA responsible	Reference
*Echinococcus granulosus* (cestode)	BMDC stimulation	Murine dendritic cells	EVs are internalised and induce maturation (CD86 upregulation)	EV proteins identified but not tested	(Nicolao et al., 2019)
*Brugia malayi* (nematode)	J774A.1 macrophage cell line	Murine macrophages	EVs are internalised and induce classically activated phenotype (increased G-CSF, MCP-1, IL-6 and MIP-2)	Proteins and RNA identified	(Zamanian et al., 2015)
*Heligmosomoides polygyrus* (nematode)	Primary macrophages (BMDM) and RAW cell line	Murine macrophages	EVs suppressed alternative and classical activation of macrophages. Suppress ST2/IL33R expression	Not investigated	(Coakley et al., 2017)
	*Alternaria* fungal allergy model in mice	Broncho-alveolar lavage, innate lymphoid cells	Reduction in lung eosinophilia, suppression of IL-5 and IL-13 in ILCs. IL-33R/ST2 suppression	Proteins and RNA identified	(Buck et al., 2014)
	MODE-K mouse cells	Intestinal epithelial cells	Dusp1 and IL-33R downregulation (MAPK signal regulator, IL-33 receptor)	miRNAs associated with Dusp1 and Il33r known	(Buck et al., 2014)
*Nippostrongylus brasiliensis* (nematode)	TNBS-induced colitis in mice	Colon tissue	EVs reduce proinflammatory cytokines IL-1β, IL-6, IL-17A and IFNγ. Increase in anti-inflammatory IL-10	Proteins/miRNAs in EVs identified but not tested	(Eichenberger et al., 2018a)
*Trichinella spiralis* (nematode, muscle larvae)	PBMC stimulation	PBMC	EVs elevate IL-6 and IL-10 production, non-significant decrease of IL-17A	Not investigated, but EVs contain immuno-modulatory proteins identified in T. spiralis ES, recognised by 7C2C5 antibody	(Kosanovic et al., 2019)
*Echinostoma caproni* (trematode)	Subcutaneous injection	Spleen	Induction of IL-4, IFNγ and TGF-β suggestive of Th2/Treg phenotype	Not investigated	(Trelis et al., 2016)
*Fasciola hepatica* (trematode)	DSS-induced colitis in mice	Not mediated by B- or T-cells (carried out in Rag1-/- mice)	EVs reduce proinflammatory cytokines TNF and IL-6, suppress neutrophil infiltration, decrease COX-2, NFκB and phosphorylation of p38 MAPK	Not investigated	(Roig et al., 2018)
*Opisthorchis viverrini* (trematode)	Immortilised human cholangiocyte culture	Human cholangiocyte	EVs promote cell proliferation and stimulate wound healing and tumorigenic pathways. IL-6 secretion	EV proteins identified but not tested	(Chaiyadet et al., 2019)
*Schistosoma japonicum* (trematode)	RAW264.7 macrophage cell line	Murine macrophages	Increased iNOS by qRT-PCR, increased TNF by qRT-PCR and ELISA Increase in surface CD16/32 by flow cytometry – skew to M1 polarisation	Not investigated	(Wang et al., 2015)

BMDC, bone marrow-derived macrophage; TNBS, 2,4,6-trinitrobenzene sulfonic acid; PBMC, peripheral blood mononuclear cell; DSS, dextran sodium sulphate; qRT-PCR, quantitative reverse transcription PCR.

There are proteins present within parasite-produced EVs that have already been characterised as immunomodulators, or with similarity to known immunomodulators. An example of this is the *H. polygyrus* protein Transforming growth factor (TGF)-β mimic, or TGM (Johnston et al., 2017). TGM was originally identified in *H. polygyrus* ES products as able to interact with TGF-β receptors and induce Foxp3^+^ T-regulatory (Treg) cells, which can then suppress effector T-cells (Johnston et al., 2017). A whole family of these molecules has since been identified, including nine further members, with five produced by the adult parasite and four by the larval stage (Smyth et al., 2018). TGM is present in *H. polygyrus* EVs (Buck et al., 2014), as well as in a soluble form that is known to potently drive Treg differentiation (Johnston et al., 2017). It remains to be determined whether vesicle-bound TGM is a more effective form of delivery that may target a wider variety of cells, and/or remain active once taken up in vesicles by host cells.

EVs from the nematode *Trichinella spiralis* are able to elevate production of IL-10 and IL-6 in peripheral blood mononuclear cells (Kosanovic et al., 2019). These EVs contain proteins recognised by the monoclonal antibody 7C2C5, which are already known to have modulatory effects on dendritic cells, translating to induction of IL-4 and IL-10 from T-cells (Cvetkovic et al., 2016). *B. malayi* EVs contain miRNAs with homology to human miRNAs, suggesting targeting of host genes (Zamanian et al., 2015). This includes the miRNA Bma-let-7 which is involved in both innate and adaptive responses to infection (Jiang, 2018). A homologue of let-7 has also been identified in *H. polygyrus* EVs, suggesting conservation of this method of immunomodulation across nematodes (Buck et al., 2014). EVs from both nematodes (*B. malayi, Teladorsagia circumcincta*) and platyhelminths (*F. hepatica, E. caproni*) contain thioredoxin peroxidases (peroxiredoxins) (Marcilla et al., 2012, Cwiklinski et al., 2015, Tzelos et al., 2016, Harischandra et al., 2018). These are proposed to be able to modulate the host immune response, including inducing development of a Th2 response via alternative activation of macrophages (Robinson et al., 2010). In addition, *B. malayi* EVs also contain macrophage migration inhibitory factor (MIF) (Harischandra et al., 2018), which can act in the same way as mouse-produced MIF to promote alternative activation of macrophages (Prieto-Lafuente et al., 2009). There is therefore a growing body of knowledge that EVs themselves represent a complex package of immunomodulators, with the vesicular structure possibly protecting the proteins within until they reach the cell in which they exert their action.

Targeting EVs to develop a vaccine against helminths is therefore a rational strategy to disable the immunomodulatory effects of the vesicles, while also priming the host immune system to detect the presence of live parasites through their release of EVs. Together, these effects seem likely to boost the immune system of the host in its efforts to clear worm infections.

## 3 Antibody interactions with EVs

Most successful vaccines act by inducing a neutralising antibody response, and protective immunity against most helminth parasites has been shown to be antibody-dependent (Hewitson and Maizels, 2014). Hence, in developing and evaluating a potential vaccine, a key parameter is generation of a potent antibody response. However, in contrast to subunit vaccines representing individual antigenic proteins, EVs present a much more complex target which may or may not offer accessible epitopes for an antibody-based vaccine strategy. In addition to the issue of whether suitable exposed antigens are present on EVs, questions are also posed of whether antibody interactions with EVs can effectively neutralise their biological functions and if such neutralisation is sufficient to lead to robust protection against infection with helminth parasites.

Evidence to date indicates that, in fact, immune recognition and neutralisation of EVs does result in significant protective immunity. Whole EVs have been used as vaccines with varying levels of success (see Table 2), ranging from nearly full clearance (Coakley et al., 2017) through to a more modest effect in reduction of egg burden (Trelis et al., 2016). Shears et al. (2018) found that EVs had to be intact to have an effect, suggesting that the configuration of immunogenic proteins, how they function and how they are displayed during a response, will have implications for a successful vaccine response.

**Table 2:** Vaccination with helminth extracellular vesicles (EVs).

Species	Vaccination method used	Results	Antibodies	Reference
*Heligmosomoides polygyrus* (nematode)	C57BL/6 mice vaccinated 3 times with EVs + alum i.p.	Vaccination decreased worm burden by 82%.	Exosomes elicited IgM, IgG1, IgA and IgE isotypes reactive with EVs. Mice vaccinated with HES or HES supernatant also generated EV-responsive IgM. Sera from EV-vaccinated mice contained both IgM and IgG1 reactive reactive to HES and HES supernatant	(Coakley et al., 2017)
*Trichuris muris* (nematode)	C57BL/6 mice vaccinated twice with EVs no adjuvant subcutaneously	Vaccination deceased worm burden by ~60%. Lysed EVs had similar results to sham control	Vaccination boosted IgG1 and IgG2a/c serum antibody responses to ES depleted of EVs. Range of IgG antibodies in sera against EV components 50–200 kDa in size. Possible targets identified	(Shears et al., 2018)
*Echinostoma caproni* (trematode)	Balb/c mice vaccinated twice with EVs no adjuvant subcutaneously	No difference in worm burden seen. Vaccination decreased EPG by ~60%. Delay in parasite development. Increase in survival rate of mice	Exosomes elicited significant IgM and IgG response in serum. IgG1, 2b and 3 subtypes responsible for IgG increase. Antibodies against exosome/ESP components mainly 90 kDa in size. Bands the same for immunisation with exosomes and infected animals. Possible targets identified	(Trelis et al., 2016)
*Opisthorchis viverrini* (trematode)	Hamsters vaccinated 3 times with EVs + alum i.p.	Vaccination decreased worm burden by 27%, EPG reduced by 32%. Average length of worms shorter	Sera showed increase in IgG against EVs both pre and post challenge. Antibodies from vaccinated hamsters blocked uptake of EVs by cholangiocytes	(Chaiyadet et al., 2019)

HES, *Heligmosomoides polygyrus* excretory/secretory products; EPG, eggs per gram.

Since the EVs themselves are not live pathogens, this outcome indicates that immunity acts by blocking EV functions such as entry into host cells, or access to target compartments within those cells, both outcomes consistent with an antibody-dependent pathway. Interestingly, in the case of *H. polygyrus* EVs, antibodies actually increase their uptake by macrophages. However, macrophages taking up EVs through the antibody-dependent pathway were protected from EV-mediated inhibition of activation (Coakley et al., 2017). Rather than accessing the host cell cytoplasm, antibody-treated EVs were directed into a degradative lysosomal pathway, thereby limiting their immunomodulatory functions (Coakley et al., 2017). Whilst enhanced EV uptake has also been shown in macrophages exposed to either *F. hepatica* EV antibodies or infected host serum, the authors of this study suggest that some specific cargo such as helminth defence molecule (HDM) and cathepsin L1 could potentially circumvent the lysosomal pathway and actually retain their modulatory function (de la Torre-Escudero et al., 2019).

In other systems such as *O. viverrini*, the role of antibodies appears to involve the inhibition of uptake, indicating that key epitopes are associated with the attachment and invasion process of the EVs (Chaiyadet et al., 2019). Although a different mechanism to directed lysosomal degradation, the net effect is similar, forestalling the ability of EVs to down-modulate host immunity. The contrast might be in the cell type targeted by EVs of different parasites, with *H. polygyrus* affecting macrophages which express Fc receptors, while *O. viverrini* interacts with cholangiocytes of the bile duct which probably do not.

A less explored question is whether, in helminth infections, the host naturally generates an antibody response to EVs and, if so, whether this contributes to acquired immunity to infection. Certainly, some EV antigens are immunogenic and in the case of cestode EVs (from *E. multilocularis* and *Taenia crassiceps*) include a range of conserved antigens known to have immunodiagnostic value, such as endophilin/p29, FABP, 14-3-3, Em18/H17g and Ts8B1 (Ancarola et al., 2017). Hence, in these infections at least, EVs must also be taken up by competent antigen-presenting cells for processing and activation of the adaptive immune response.

## 4 Vaccine antigens

Although vaccination with native EVs isolated from helminth ES products has served as a useful proof of concept, it will not be feasible to scale up as a clinical vaccine for general use. In addition, it will be important to define vaccine target molecules to evaluate efficacy of antibody generation in different populations, to research possible antigenic variation in parasite isolates, and to monitor any future loss of protection. For these reasons, attention is now turning to identifying and testing specific EV-associated antigens which can be formulated into a vaccine that recapitulates the protective action of vaccination with whole EVs.

In principle, the EV represents a large membranous surface which envelops a relatively small intravesicular volume (Buzas et al., 2018); it is to be expected therefore that a high proportion of EV-associated proteins will be linked to the vesicular membrane, although one cannot necessarily predict which may be exposed on the exterior surface. In mammalian EVs, several hundred membrane proteins have been characterised, including highly conserved proteins such as tetraspanins (CD9, CD63 and CD81) with two extracellular loops separated by four transmembrane segments, as well as proteins involved in cell adhesion, and a variety of ectoenzymes, any or all of which may be required for the process of target cell entry. While some EV surface components are integral membrane proteins, others may be tethered by those membrane proteins, as for example in TGF-β attached to the betaglycan receptor (TβRIII) on cancer exosomes (Webber et al., 2010).

Many similar proteins have been identified in helminth EVs. Tetraspanins appear to be a defining feature of both host and parasite EVs (Andreu and Yanez-Mo, 2014), and notably had been flagged as potential vaccine antigens in a number of earlier studies. In *S. mansoni*, both TSP-1 and TSP-2 induce a protective response in mouse models, TSP-2 more so, and this protein also elicited strong antibody responses in exposed but uninfected residents of an endemic area in Brazil (Tran et al., 2006). Sm-TSP-2 has been further developed as a lead candidate for a human *S. mansoni* vaccine (Cheng et al., 2013). However, in *Schistosoma japonicum*, TSP-2 was less effective and showed considerable polymorphic variation between isolates, potentially limiting its application as a vaccine target (Zhang et al., 2011). Seven TSP proteins from *E. multilocularis* have also been tested in a mouse model, using the larger extracellular loop, with encouraging results in particular for Em-TSP-3 (Dang et al., 2009, Dang et al., 2012). Similarly, TSP from the human filarial nematode *B. malayi* showed protective effect in a mouse model (Dakshinamoorthy et al., 2013). However, tetraspanins are also found on the external surface of each of these parasites, and hence it is not established whether TSP vaccination acts primarily against EVs, or directly against the worms themselves.

As well as conserved and probably functionally essential components such as tetraspanins, EVs contain a suite of products derived from their parental cells. In the case of *H. polygyrus* EVs, many proteins associated with the apical membrane of the nematode intestinal tract were identified; electron microscopy also visualised vesicular bodies in the lumen of the worm gut, and some multi-vesicular bodies in the underlying tissue (Buck et al., 2014). In addition, EVs from this parasite contained ectoenzymes such as the metalloproteases (MEPs) and H11 (Buck et al., 2014). *Heligmosomoides polygyrus* is closely related to the trichostrongyloid parasites of sheep such as *Haemonchus contortus* (the barber's pole worm), and it is striking that H11 and MEPs have been studied for many years as leading vaccine antigens against this parasite (Andrews et al., 1995, Newton et al., 1995); indeed a H11/MEP combination is now commercially available as “Barbervax” and is comprised of native membrane glycoproteins purified from parasite extracts that is effective in sheep (Smith et al., 2001). Although classically, the mode of action has been assumed to be antibody interference with intestinal function in the worm, the possibility should now be considered that vaccine-induced antibodies block EVs from this parasite from executing their immunomodulatory function.

Whilst treatment with Barbervax is shown to confer protection against *H. contortus* infection, it fails to elicit a long-term memory response. As H11 and MEPs are parasite gut antigens, they are largely “hidden” from the host during infection and fail to induce natural immunity, thus frequent vaccination boosts might be required in order to maintain protection. This is an important consideration when developing future helminth vaccines that may utilise EV-associated antigens. Ideally, vaccines which are generated against either molecules exposed on the EV surface, or EV-derived epitopes which have been processed and presented during an adaptive immune response may have a greater chance of long-term protective responses in endemic areas.

To date, there has been little systematic research into the mode of action of EV-associated proteins. Many important issues should be addressed, in particular the relation of each different stage of parasite to the target antigen, establishing for example if a vaccine would act on immature or migratory larvae, or only on the mature adult stage. The question of EV heterogeneity also remains to be investigated, as it is currently unknown if expression of each vaccine antigen is universal, and if not, whether subsets of EVs that escape immune attack can still act in an immunomodulatory fashion. Finally, if conserved EV antigens are to be targeted, caution is required where similar proteins are also expressed in the mammalian host in the event that vaccination would interfere with host physiological processes, or indeed prompt an autoimmune response.

## 5 Vaccine delivery

In parallel with identifying effective helminth antigens, it is also important to consider their means of delivery for future vaccines. In fact, extracellular vesicles may have intrinsic properties that promote immune responses, as in the case of outer membrane vesicles from Gram-negative bacteria that present multiple Toll-like receptor (TLR) ligands for innate immune activation (Tan et al., 2018). Vesicles can be loaded with exogenous antigens, or harvested from bacteria expressing recombinant parasite proteins; where antigens are encapsulated within the lipid bilayer, they would be protected from degradation in the extracellular space, might be released in a measured fashion over time, thereby maximising immunogenicity. As well as manipulated EVs, it is also possible to create synthetic liposomes as both antigen and adjuvant carriers (Nisini et al., 2018), and as recently reported, a recombinant *E. granulosus* antigen/liposome complex stimulated protective immunity of 95% in mice (ZongJi et al., 2019).

Mammalian-derived exosomes also pay critical roles in immune regulation and activation (Thery et al., 2009), with dendritic cell (DC)- and tumour-derived exosomes found to induce eradication of tumours (Syn et al., 2017). Exosomes from DCs pulsed with *Toxoplasma gondii*-derived antigens were able to produce protective responses against infection and congenital toxoplasmosis in mice (Beauvillain et al., 2007, Beauvillain et al., 2009), showing that this method might also be effective for parasites. The success of using exosomes derived from DCs is believed to be due to the presence of functional MHC class I and II and T-cell costimulatory molecules on their surface which can display antigen to the immune system (Thery et al., 2002). Some form of antigen presentation via a receptor on the surface may therefore be required for an EV-based helminth vaccine to be successful. Another important consideration in utilising DC-derived EVs is the heterogeneity of the vesicles produced, as Wahlund and colleagues were able to demonstrate distinct differences in the antigen processing and immunostimulatory capacity of microvesicles and exosomes in vivo (Wahlund et al., 2017). Directed targeting of an antigen to the exosome surface has been found to improve immunogenicity of adenoviral vaccines in mice (Bliss et al., 2020), so a more convenient means of vaccination could be to drive the host to secrete the antigens within its own EVs, rather than manufacturing EVs with the correct configurations for antigen presentation.

Packaging into vesicles also makes mucosal delivery of the antigen possible, as lipid-based particles can protect the contents from degradation (Corthesy and Bioley, 2018). Mucosal delivery has been found to be more effective at inducing antigen-specific immune responses at mucosal surfaces and is found to be just as effective as parenteral administration at inducing systemic immune responses (Lycke, 2012). This would be ideal in combatting helminth infection, which often takes place at or involves mucosal sites. Oral vaccination using a *N. americanus* lipopeptide in both nanoparticles or packaged in liposomes has been demonstrated to be effective in the *N. brasiliensis* mouse model (Bartlett et al., 2020). Mucosal vaccination, for instance through oral or nasal routes, would also offer the advantage of ease of administration, making mass vaccination possible in the least developed and developing countries where these helminth infections are endemic.

## 6 Concluding remarks

The possibility of using EVs, and EV antigens, as novel helminth vaccines is a new and exciting entrant into the field of parasitology. As discussed above, a myriad of possibilities now need to be explored, ranging from the identification of individual antigens, the elucidation of mode of action, and the optimisation of vaccination regimens. We have as yet little information on how best to present EV antigens, or indeed how best to express them or accompany them with the most suitable adjuvant, and still no insight into the most effective mode of immune response that a vaccine would seek to induce. However, much progress continues to be made at the more empirical level, validating choice of key antigens and providing proof-of-principle for the global approach is now under way. We look forward to many more incisive findings in both detailed reductionist analysis, and system-wide immune outcomes that may together deliver a ground breaking vaccine strategy for helminth diseases.
